# Surgical Management of Proximal Humerus Fractures in Patients With Common Injury-Specific Comorbidities

**DOI:** 10.7759/cureus.15203

**Published:** 2021-05-23

**Authors:** Blake Callahan, Batool Zehra

**Affiliations:** 1 Medicine, University of Central Florida College of Medicine, Orlando, USA; 2 Internal Medicine, Naples Community Hospital, Naples, USA

**Keywords:** proximal humerus fracture, alcohol withdrawal syndrome, osteoporosis, open reduction internal fixation, scapula and shoulder trauma, surgical management

## Abstract

When evaluating a humeral neck fracture for surgical intervention, it is prudent to evaluate the patient for the common injury-specific comorbidities of alcoholism and osteoporosis, as the presence of either of these conditions, require additional considerations to prevent complications.

This case presents a 63-year-old female who presented for evaluation after multiple falls. She was discharged from an outside facility one week prior with a left (nondominant extremity) humeral neck fracture. Her condition was complicated by alcohol use disorder with recent heavy alcohol use. On hospital stay day one, orthopedic surgery performed an initial assessment and deemed her fracture to be nonoperative due to medical comorbidities. On hospital stay day three, the patient requested a second opinion from orthopedic surgery due to continued increased pain and concern for the long-term function of her left upper extremity. The second opinion found that the fracture would be best managed by surgical intervention now that she had been medically optimized since admission. On hospital stay day five, she was taken to the operating room for planned percutaneous intramedullary nail placement with closed reduction of the fracture. Intraoperatively, the percutaneous procedure had to be converted to an open procedure due to the difficult nature of her osteoporotic bone in terms of performing a closed reduction. This case discusses the various methods for surgical management and guidelines for optimizing patients with the fracture-specific common comorbidities of alcohol use disorder and osteoporosis. The novelty of this case includes the rarity of a patient with both the major predisposing factors to proximal humerus fractures and includes a literature review of the latest recommendations for surgical management.

## Introduction

The proximal humerus fractures are commonplace in two subsets of patients - patients with alcohol use disorder, regardless of age, and elderly patients with osteoporosis. Both patient populations present unique challenges when considering the surgical management of proximal humerus fractures. For alcohol use disorder, the challenges consist of preventing complications from withdrawal and poor wound healing [1]. Osteoporosis presents with further challenges of requiring more complicated fracture fixation in addition to the possibility of inappropriate bone quality leading to re-fracture or failure of the bone to incorporate into hardware [2,3]. Given these common comorbidities that often coincide with proximal fractures, orthopedic surgeons must be vigilant, both preoperatively and intraoperatively, when caring for such patient populations.

## Case presentation

A 63-year-old female presented to the emergency department after an unwitnessed fall and was found to have a severely displaced left (dominant extremity) proximal humerus fracture. She had previously presented to an outside hospital one week prior after a fall and was found to have a left shoulder humeral fracture at that time and was discharged home.

She explained that these falls had been occurring for more than six months and initially started as episodes with an activity where she would feel dizzy and blackout; she noted that she frequently injured herself in the process. Over time, these falls became more frequent, and she had difficulty explaining the events leading to her more recent falls. She had a past medical history significant for alcohol use disorder, recurrent falls, post-traumatic stress disorder, panic disorder, anxiety, and depression. She had no known surgical history. 

On physical examination, her left shoulder had visible diffuse and a large amount of ecchymosis present from proximal shoulder extending into the hand. There was moderate swelling in her left arm, forearm, and hand. Her radial pulse was 2+. Range of motion testing was deferred due to intractable pain from the fracture. Her neurologic examination consisted of her being alert and oriented to person, place, time, and situation. Left upper extremity sensation was intact to light touch. The patient was cooperative, responded to questions appropriately, and had an appropriate mood and affect. The rest of the physical examination was unremarkable.

Objective data

The initial vitals on presentation were - temperature 98.5°F, weight 108 lbs, heart rate 75 beats per minute, respiratory rate 18 breaths per minute, blood pressure 100/70 mmHg.

The patient was found to have significant laboratory tests, including anemia with a hemoglobin of 8.9 g/dL, hypokalemia with potassium of 2.9 mEq/L, hypochloremia with chloride of 93 mEq/L, hypomagnesemia with magnesium of 1.4 g/dL, elevated lipase of 1,223 U/L, elevated liver function enzymes including alanine transaminase (ALT) of 229 U/L, aspartate aminotransferase (AST) of 517 U/L, and lactic acidosis with a lactic acid level of 1.8 mmol/L. Electrocardiogram (EKG) indicated normal sinus rhythm. Serially trended troponins were not elevated. The urinary drug screen was negative. The serum ethanol screen was negative.

**Figure 1:**
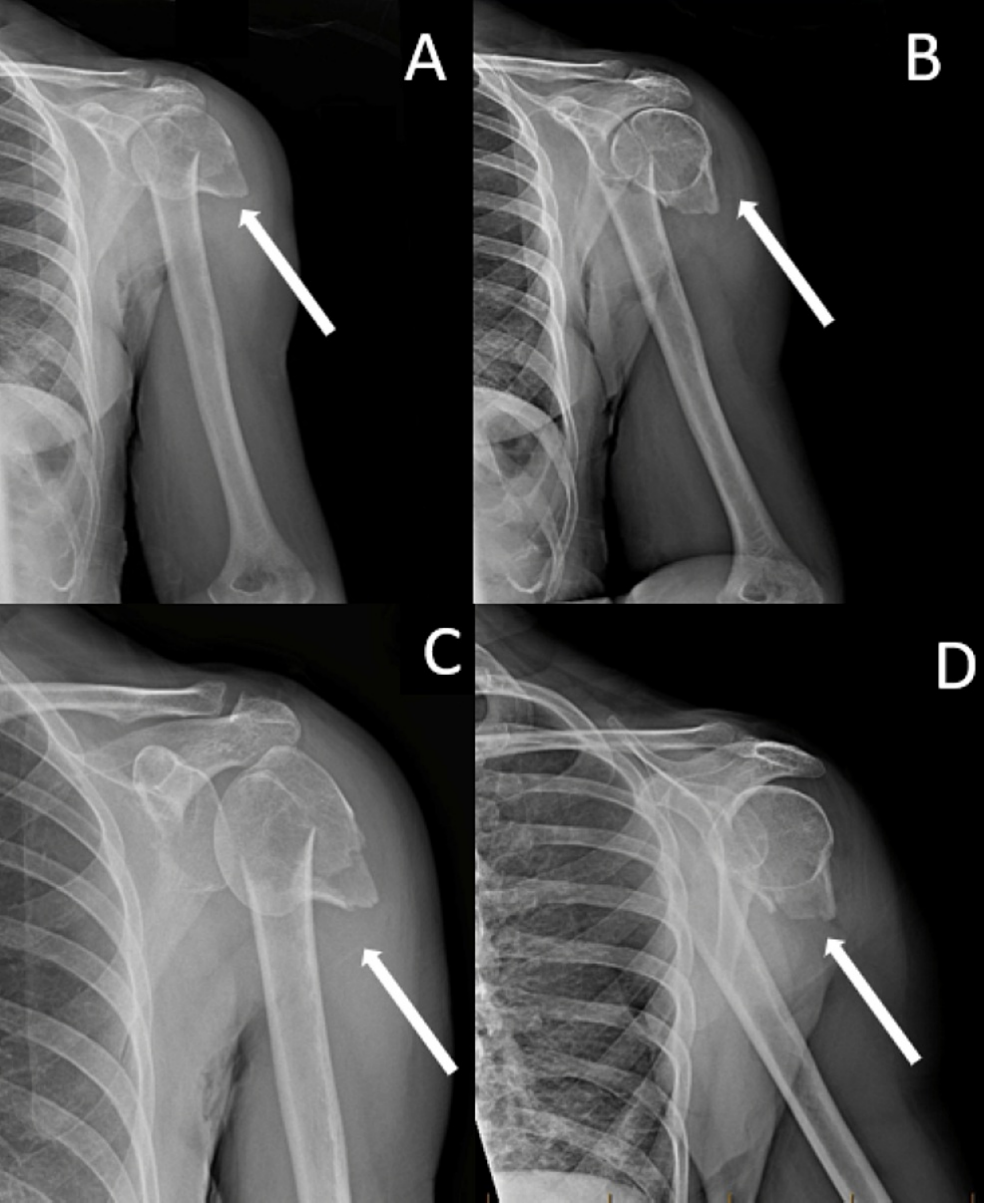
Anteroposterior and Lateral X-Ray Views of the Left Humerus and Shoulder Anteroposterior (AP) (A) and lateral (B) x-ray views of the left humerus and AP (C) and lateral (D) x-ray views of the left shoulder demonstrate a two-part left proximal humeral neck fracture with severe displacement and angulation between the proximal humeral head and distal humeral fragments.

CT chest, abdomen, and pelvis were unremarkable. Left-hand x-ray showed soft tissue swelling. Left forearm x-ray showed soft tissue swelling. Left humerus x-ray showed acute displaced humeral neck fracture as demonstrated in Figure 1.

After an initial assessment, the patient received 2 g of magnesium sulfate, 40 mEq of potassium chloride, and a 500 mL bolus of normal saline with supplementation of a banana bag to correct her multiple electrolyte abnormalities. Her left arm was wrapped in an ace bandage and placed in a shoulder immobilizer. The patient was placed in the Clinical Institute Withdrawal Assessment for Alcohol (CIWA) protocol and a diet directed for nothing to eat or drink (NPO) pending evaluation from orthopedic surgery.

Orthopedic surgery was consulted for the evaluation of left humerus fracture and after evaluation decided to proceed with nonoperative management due to the patient’s elevated fall risk and risk for delirium tremens. The patient continued to complain of severe pain despite pain control modalities. She was concerned that her shoulder would not heal properly, and she would not be optimally functional with the left upper extremity if conservative management was continued. On hospital stay day three, at the patient’s request, a second opinion from orthopedic surgery was obtained. At that point, the surgical team believed her to be medically optimized and outside the window of greatest concern for alcohol withdrawal complications. Surgical management was planned accordingly.

Operative course

**Figure 2:**
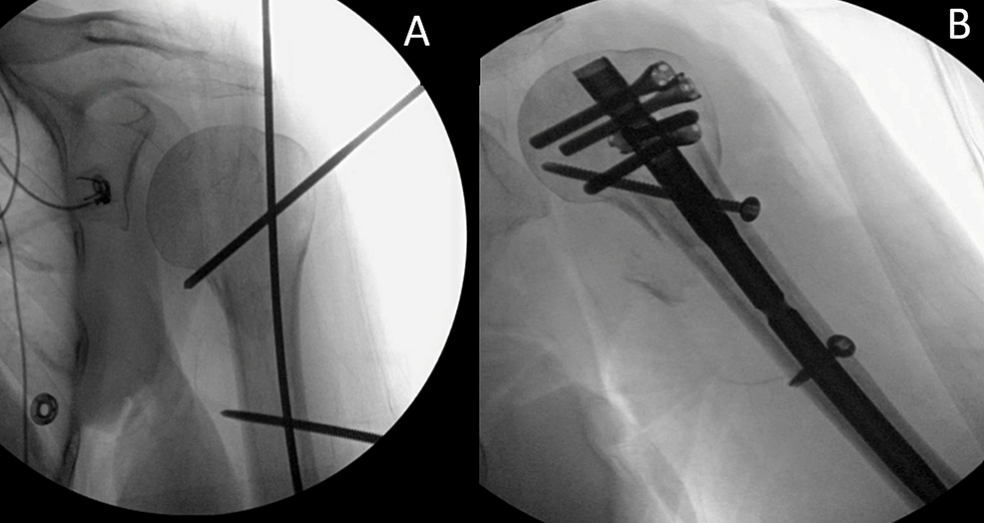
Intraoperative Fluoroscopic Images of the Left Shoulder Intraoperative fluoroscopic images of the left shoulder (A and B) demonstrate proper reduction of the humeral neck fracture with the return to normal anatomic position and fixation with intramedullary screw, additional screws for structural support, and distal locking screw.

On hospital stay day five, the patient was taken to the operating room for a planned intramedullary nailing procedure of proximal humerus fracture. Intraoperatively, two Kirschner wires were placed, one just inferior to her fracture and a second-placed from the lateral to the medial portion of her humerus; they were used as joysticks to attempt the reduction of the fracture. After prolonged attempts at closed reduction, the decision was made to convert the procedure to an open fashion. The identified bony fragments in the fracture were turned into anatomical positions. This allowed direct open reduction of the fracture. A guidewire was passed from the top of the humerus into the humeral canal and an intramedullary nail was impacted once the fracture was held in appropriate apposition. Proximal screws were placed, and osteoporotic bone was noted so additional screws were added to stabilize the construct. Intraoperative x-rays demonstrating proper reduction of the fracture and placement of the intramedullary nail are shown in Figure 2. The patient was discharged to inpatient rehabilitation on a postoperative day four without complications.

## Discussion

When surgical management of proximal humerus fractures is warranted, there are several surgical approaches available to achieving reduction and fixation of the fracture. Such methods include open reduction and internal fixation with screws and locking plates, intramedullary nailing, hemiarthroplasty, and even reverse total shoulder arthroplasty [2]. Each patient’s medical comorbidities should be evaluated before considering if a patient is a candidate for surgery. Further consideration should be taken to determine which surgical procedure is most beneficial. In this case, the decision to pursue surgical management was complicated by the patients underlying medical comorbidities. Unfortunately, these medical comorbidities are incredibly common in persons with humerus fractures. 

One common comorbidity found in patients with humerus fractures is alcohol use disorder. One study concluded that around 12% of all shoulder injuries in those aged between 34 years and 64 years old were found in alcohol abusers and nearly half of proximal humerus fractures were found in this population [4]. Alcohol use can drastically impact both perioperative management and postoperative care. The most concerning aspect of alcohol use disorder in surgical candidates is the possibility of delirium tremens. Delirium tremens is a condition that occurs six to 48 hours after the last alcoholic beverage and is a direct result of the alcohol withdrawal process. It can potentially cause life-threatening seizures and cardiovascular collapse [1].

To prevent withdrawal complications, one must first assess whether a patient is at risk of withdrawal by assessing alcohol use. The patient-reported assessments typically include methods such as the CAGE Substance Abuse Screening Tool and Alcohol Use Disorders Identification Test (AUDIT) but are limited to the willingness of patients to accurately provide information [1]. Laboratory abnormalities that should raise suspicion for alcohol abuse include an increased mean corpuscular volume (MCV), an AST to ALT ratio of greater than 2:1, elevated lipase levels, increased gamma-glutamyltransferase (GGT) levels, and positive urine alcohol tests.

Once identified for being at risk for alcohol-related complications patients should be medically managed for prevention and reduction of withdrawal symptoms. The Clinical Institute Withdrawal Assessment for Alcohol Scale (CIWA) protocol is a trial validated method for both assessing and directing the use of therapeutic modalities [4]. Long-acting benzodiazepines particularly lorazepam or diazepam are mainstays in treatment with alternatives including α2-agonists such as clonidine or even continued oral or enteral alcohol administration [1].

In the perioperative setting, Ungur et al. suggest an algorithm based on AUDIT screening scores to guide surgical prophylactic and intraoperative medical management [1]. Anesthesiology should take further intraoperative considerations regarding cross-tolerance of alcohol with anesthetic and analgesic medications potentially requiring dose adjustments from persons without alcohol use disorder.

Another comorbidity heavily correlated with proximal humerus fractures is osteoporosis [2]. Poor bone quality is a known predictor of intraoperative and postoperative complications; therefore, if a patient with known osteoporosis requires surgical intervention, careful considerations must be taken. The potential complications include failure to achieve perfect reduction, the need for additional screws for fixation, as well as avascular necrosis and screw cutout (where the areas around screws fail to grow in, there is loss of surrounding bone, potential local collapse, loss of reduction, and screw displacement) [3]. Given these concerns, it is appropriate for orthopedic surgeons to assess bone quality preoperatively in patients at risk for osteoporosis. Such preoperative evaluation methods typically include nuclear or radiographic measures including the use of dual-energy x-ray absorptiometry (DEXA) scans, peripheral quantitative computed tomography (pQCT), and deltoid tuberosity index measurements [3]. One study suggests the use of the deltoid tuberosity index is the most reliable and readily available method for bone mineral density prediction [3]. This is a radiographic measure of the bone cortex to medullary cavity thickness at the level of the deltoid tuberosity in an anterior to posterior x-ray view of the humerus. The suggested deltoid tuberosity index (DTI) cutoff value with the highest sensitivity and specificity for low bone mineral density (defined as <80 mg/cm^3^) is 1.4 [3].

When patients meet the criteria for osteoporosis, the literature suggests that certain surgical techniques may be superior to others in terms of outcomes. Intramedullary nails may be appropriate for two-part fractures. Plate fixation is an alternative to intramedullary nailing. Hemiarthroplasty (if adequate supraspinatus function) or reverse total shoulder arthroplasty is required when there is suspected vascular compromise of the humeral head or if fractures are not candidates for reduction and fixation techniques [5].

When attempting plate or intramedullary nail fixation, best practices include achieving the best possible reduction of the tuberosities while buttressing the metaphyseal borders to achieve structural support of osteoporotic bone. One method that is considered to have favorable outcomes in those with osteoporosis is reverse total shoulder arthroplasty (RTSA). RTSA is now the most often used approach for patients with severe osteoporosis and takes place in approximately two-thirds of patients that undergo arthroplasty [6].

Regardless of the surgical approach, some techniques may be used to augment the osteoporotic bone to increase construct strength and reduce complications. Some suggested methods of augmentation include the addition of intramedullary fibular grafts, calcium phosphate cement, iliac crest bone grafts, plate fixation with heavy, nonabsorbable sutures through the rotator cuff, and construct fixation sutures [5]. Additional medial support screws can offer support if the medial cortex is compromised. 

## Conclusions

This case highlights the comorbidities of alcohol use disorder and osteoporosis that are commonplace in patients that present with humerus fractures. These conditions should be actively screened for when a humeral fracture is revealed. If evaluated properly, considerations can help prevent and manage surgical complications and can lead to the best clinical outcomes.
